# Improved lung cancer clinical outcomes in patients with autoimmune rheumatic diseases

**DOI:** 10.1136/rmdopen-2023-003471

**Published:** 2023-10-31

**Authors:** Paola Ghanem, Joseph C Murray, Kristen A Marrone, Susan C Scott, Josephine L Feliciano, Vincent K Lam, Christine L Hann, David S Ettinger, Benjamin P Levy, Patrick M Forde, Ami A Shah, Christopher Mecoli, Julie Brahmer, Laura C Cappelli

**Affiliations:** 1 Department of Oncology, The Johns Hopkins University School of Medicine, Baltimore, Maryland, USA; 2 Division of Rheumatology, Department of Medicine, The Johns Hopkins University School of Medicine, Baltimore, Maryland, USA

**Keywords:** Epidemiology, Autoimmune Diseases, Autoimmunity

## Abstract

**Purpose:**

Concomitant autoimmune rheumatic diseases (ARD) can add morbidity and complicate treatment decisions for patients with lung cancer. We evaluated the tumour characteristics at diagnosis and clinical outcomes in lung cancer patients with or without ARD.

**Methods:**

This retrospective cohort study included 10 963 patients with lung cancer, treated at Johns Hopkins. Clinical data including tumour characteristics and outcomes were extracted from the cancer registry. Data on patients’ history of 20 ARD were extracted from the electronic medical record. Logistic regression was used to compare tumour characteristics between those with and without ARD; Kaplan-Meier curves and Cox proportional hazards models were performed to compare survival outcomes.

**Results:**

ARD was present in 3.6% of patients (n=454). The mean age at diagnosis was 69 (SD 10) and 68 (SD 12) in patients with and without ARD (p=0.02). Female sex and smoking history were significantly associated with a history of ARD (OR: 1.75, OR: 1.46, p<0.05). Patients with ARD were more likely to be diagnosed with stage 1 lung cancer (36.8% vs 26.9%, p<0.001) and with smaller tumour size (OR: 0.76, p=0.01), controlling for sex, race and histology. Notably, lung cancer patients with ARD had a significantly prolonged median overall survival (OS) (7.11 years vs 1.7 years, p<0.001), independent of stage.

**Conclusion:**

Patients with ARD and lung cancer had better OS compared with their counterparts, independent of cancer stage and treatments and were less likely to have advanced stage lung cancer at diagnosis. Additional studies are needed to investigate the differential immunological anti-tumour immune activity and genomic variations in patients with and without ARD.

**WHAT IS ALREADY KNOWN ON THIS TOPIC**

- Certain autoimmune rheumatic diseases such as myositis and scleroderma have been associated with increased risk for malignancy. It is unclear whether autoimmune disease worsens, enhances, or does not affect antitumour immune responses and clinical outcomes in lung cancer.

**WHAT THIS STUDY ADDS**

- This study shows that autoimmune rheumatic disease is associated with earlier stage lung cancer at diagnosis and better clinical outcomes for lung cancer.

**HOW THIS STUDY MIGHT AFFECT RESEARCH, PRACTICE OR POLICY**

- This study prompts follow-up translational evaluation of potential immunological underpinnings of differences in lung cancer presentation and outcomes in autoimmune disease.

## Introduction

Autoimmune rheumatic diseases (ARD) are a heterogenous group of disorders that can significantly affect morbidity and mortality.^1^ About 3%–5% of the general population is affected by an autoimmune disease.^1^ The epidemiology of an individual ARD varies significantly by age, sex, ethnicity and other clinical features. Previous studies have identified an increased cancer risk in patients with ARD potentially due to an underlying dysregulated immune system,^2^ a pro-inflammatory chronic state and impaired regulatory T cells.^3 4^ In particular, an increased incidence rate of cancer in patients with dermatomyositis, polymyositis and scleroderma has been observed.^5^ Rheumatoid arthritis (RA) has also been found to be associated with an increased risk of lymphoma and lung cancer.^6^ Previous studies have established shared risk factors among rheumatic diseases and malignancies. This is particularly true when it comes to smoking status, as patients with RA experience an increased incidence of smoking associated cancers,^7^ as well as alcohol use^8 9^ and obesity.^10^ Understanding the association between cancer development in patients with autoimmune disease is of key importance both in better defining the pathogenesis of autoimmunity and in understanding how autoimmune diseases may influence cancer outcomes in this subpopulation.

Lung cancer remains the leading cause of cancer-related death worldwide.^11 12^ Additionally, lung cancer is enriched in patients with autoimmune diseases with a mean standardised incidence ratio of 1.33.^2^ Given the prevalence of lung cancer and the association between lung cancer and autoimmune diseases like RA, evaluating clinical outcomes of lung cancer patients with ARD is of particular clinical importance. While a limited number of previous studies have investigated the clinical outcomes of cancer patients with ARD, there remain conflicting evidence on comparative clinical outcomes in this group of patients.^13–15^ From the standpoint of pathogenesis, it is also not clear whether autoimmune disease worsens, enhances or does not affect antitumour immune responses. Further complicating treatment of patients with ARD and cancer in recent years is the increased use of immunotherapy which can cause flares of the underlying ARD.^16 17^ Overall, an improved understanding of the relationships between cancer and ARD could improve clinical care, guide disease monitoring and increase knowledge of the biological relationship between these two entities. In this study, we assessed the tumour characteristics at diagnosis and clinical outcomes among lung cancer patients with or without ARD in a large retrospective cohort.

## Methods

### Study design and patient selection

We performed a retrospective observational cohort study to investigate the clinical outcomes of lung cancer patients with and without ARD. Patients were 18 years of age or older and were diagnosed with lung cancer of any histology from 2004 to 2021. Patients who did not receive their first course of cancer treatment at Johns Hopkins or had missing data on the presence of ARD (eg, no electronic medical records with medical history or problem list available) were excluded from the study.

### Study outcome and covariates

Clinical data were abstracted from the institution’s prospectively collected cancer registry and included patients’ demographic features (age at diagnosis, sex, race, ethnicity, alcohol history, smoking history), tumour and treatment characteristics (histology, stage, grade, distant metastatic sites, treatment modalities) and clinical outcomes (vital status, overall survival (OS)). Treatment modalities included surgery, radiation therapy, immunotherapy and chemotherapy.

The registry’s ‘immunotherapy treatment’ modality included various classes of monoclonal antibodies such as immune checkpoint inhibitors as well as other monoclonal antibodies such as bevacizumab, trastuzumab and others as reflected in the Standards for Oncology Registry Entry Manual.^18^ The radiation modality includes radiation therapy to the primary tumour or draining lymph node regions associated with the tumour and includes external beam, brachytherapy or a radioisotope. Disease staging (1–4) was determined by the American Joint Commission on Cancer and International Union Against Cancer (version 8) tumour, node, metastases criteria. Tumours’ histology belonged to one of the following: adenocarcinoma, squamous cell carcinoma (SCC), small cell lung cancer (SCLC) and non-SCLC (NSCLC)-not otherwise specified (table 1).

**Table 1:** Demographic and clinical characteristics by autoimmune disease status

		No ARD N=10 561 (%)	ARD N=402 (%)	P value
Age at diagnosis (mean years±SD)		67±12	69±10	0.02
Sex N=10 941	Male	5096 (48.3)	145 (20)	<0.001
	Female	5444 (51.7)	256 (80)	
Race N=10 840	White	7754 (74.3)	282 (70.5)	0.001
	Black	2072 (19.8)	94 (23.5)	
	Native American	4 (0.0)*	2 (0.5)*	
	Asian	473 (4.5)	15 (3.8)	
	Other	137 (1.3)	7 (1.8)	
Alcohol history N=6954	None	3190 (48.1)	184 (58.2)	0.002
	Current	2808 (42.3)	108 (34.2)	
	Former	640 (9.6)	24 (7.6)	
Tobacco history N=7503	Never	1119 (15.6)	45 (12.9)	0.001
	Current	2324 (32.5)	88 (25.1)	
	Former	3710 (51.9)	217 (62)	
Histology N=10 963	Adenocarcinoma	5300 (50.2)	206 (51.2)	0.2
	SCC	1886 (17.9)	76 (18.9)	
	NSCLC-NOS	1812 (17.2)	65 (16.2)	
	SCLC	1109 (10.5)	31 (7.7)	
	Large neuroendocrine	454 (4.3)	24 (6.0)	
Clinical stage† N=9249	Stage 1	2388 (26.9)	131 (36.6)	<0.05
	Stage 2	631 (7.1)	43 (12)	
	Stage 3	1899 (21.4)	74 (20.7)	
	Stage 4	3973 (44.7)	110 (30.7)	
Clinical T stage‡ N=8505	T0–T2	4957 (60.8)	234 (67.6)	0.01
	T3–T4	3202 (39.2)	112 (32.4)	
Bone metastasis N=7385	No	7019 (100)	366 (100)	NA
Liver metastasis N=7380	No	6408 (91.4)	345 (94)	0.07
	Yes	605 (8.6)	22 (6)	
Brain metastasis N=1285	No	6097 (86.9)	324 (93.2)	<0.001
	Yes	918 (13.1)	25 (6.8)	
Surgery N=10 963	No	6804 (64.4)	207 (51.5)	<0.001
	Yes	3757 (35.6)	195 (48.5)	
Chemotherapy N=10 963	No	4268 (40.4)	179 (44.5)	0.09
	Yes	6293 (59.6)	223 (55.5)	
Immunotherapy/monoclonal antibodies N=10 963	No	9603 (90.9)	322 (80.1)	<0.001
	Yes	958 (9.1)	80 (19.9)	
Radiation therapy N=10 963	No	7293 (69.1)	298 (74.1)	0.031
	Yes	3268 (30.9)	104 (25.9)	

*Statistically different distribution among both groups.

†Clinical stage is subcategorised into early representing stage 1 and 2 and late-stage representing stage 3 and 4.

‡T stage describes the size of the tumour and any spread of cancer into nearby tissue; defined by the eight-edition stage of the American Joint Commission on Cancer.

AD, autoimmune disease; ARD, autoimmune rheumatic diseases; NOS, not otherwise specified; NSCLC, non-small cell lung cancer; SCC, squamous cell carcinoma; SCLC, small cell lung cancer.

**Table 2:** Clinical features associated with autoimmune disease in patients with lung cancer

	OR	95% CI	OR adjusted	95% CI
Sex (reference: male)				
Female	1.65*	1.34 to 2.03	1.53*	1.19 to 1.98
Race (reference: white)				
Black	1.25	0.98 to 1.58	1.10	0.83 to 1.46
American Indian	13.75*	2.51 to 75.37	13.75*	2.21 to 85.43
Asian	0.87	0.51 to 1.48	0.46	0.20 to 1.07
Smoking history (reference: never)				
Current/former	1.26	0.91 to 1.73	1.22	0.84 to 1.77
Alcohol history (reference: never)				
Current/former	0.64	0.53 to 0.83	0.70*	0.54 to 0.90
Histology (reference: adenocarcinoma)				
SCC	1.04	0.79 to 1.36	1.05	0.76 to 1.45
NSCLC-NOS	0.92	0.69 to 1.23	0.97	0.68 to 1.39
SCLC	0.72	0.49 to 1.05	0.65	0.39 to 1.07
Large neuroendocrine	1.36	0.88 to 2.10	1.19	0.68 to 2.08
Clinical stage (reference: stage 1)				
Stage 2†	1.24	0.87 to 1.77	1.44	0.96 to 2.16
Stage 3	0.71*	0.53 to 9.95	0.85	0.60 to 1.18
Stage 4	0.51*	0.39 to 0.65	0.58*	0.42 to 0.78

*Statistically significant.

†Advanced cancer was defined as stage 3 or 4.

NOS, not otherwise specified; NSCLC, non-small cell lung cancer; SCC, squamous cell carcinoma; SCLC, small cell lung cancer.

Clinical data on patients’ medical history of ARD was extracted from the electronic medical record for each patient identified from the cancer registry by an experienced EPIC CCDA Adjunct Analyst. The search used ICD-10 codes to evaluate the presence of ARD, listed in online supplemental table 1. Patients’ ARD status was validated by manually searching patients’ charts to confirm the history of ARD in a randomly selected subset of 5% of patients. A total of 20 ARD were investigated in this study: antisynthetase syndrome, autoimmune necrotising myopathy, dermatomyositis, polymyositis, RA, lupus/systemic lupus erythematosus, antiphospholipid antibody syndrome, psoriatic arthritis, psoriasis, ankylosing spondylitis, spondyloarthropathy, undifferentiated inflammatory arthritis, scleroderma/systemic sclerosis, scleromyxedema, vasculitis (cerebral, cryoglobulinaemia, cutaneous, hypocomplementemic urticarial/rheumatoid, IgA, large vessel, leucocytoclastic vasculitis, granulomatosis with polyangiitis, polyartheritis nodosa, microscopic polyangiitis, eosinophilic granulomatosis with polyangiitis), polymyalgia rheumatica, Sjogren’s syndrome, undifferentiated connective tissue disease, mixed connective tissue disease, gout/pseudogout. Psoriatic disease was recoded to include psoriatic arthritis and psoriasis. Myopathy included patients diagnosed with either dermatomyositis or polymyositis. Missing data for these covariates was rare and did not exceed 5% of the cohort. We therefore performed a complete case analysis.

10.1136/rmdopen-2023-003471.supp1 Supplementary data

### Statistical analysis

Descriptive statistics were calculated for the total, ARD group and non-ARD group and were expressed as mean±SD for continuous variables and count (per cent) for categorical variables. χ^2^ test was used to evaluate the association between the clinical, patient and tumour characteristics and the history of ARD. Independent t-test was used to evaluate the mean difference in continuous variables among lung cancer patients with and without ARD. We conducted multivariate logistic regression of tumour characteristics in those with and without ARD accounting for clinically meaningful characteristics. Kaplan-Meier curves and Cox proportional hazards models were performed to compare the survival outcomes (OS) of lung cancer patients with and without ARD, adjusting for age, cancer stage and the different treatment modalities. Because our study was not powered to assess the clinical outcomes of patients with lung cancer within specific subcategories of ARD, we present the clinical and survival analyses conducted in each subcategory of autoimmune diseases that had at least 20 cases in online supplemental tables 2 and 4. To further, increase our power, we grouped ARDs with similar immunological backgrounds together and present the clinical and survival analyses in online supplemental tables 3 and 5. All p values were based on two-sided testing and differences were considered significant at p value <0.05. All statistical analyses were conducted using IBM Statistical Package for Social Sciences V.27.

## Results

### Cohort characteristics

**Figure 1:**
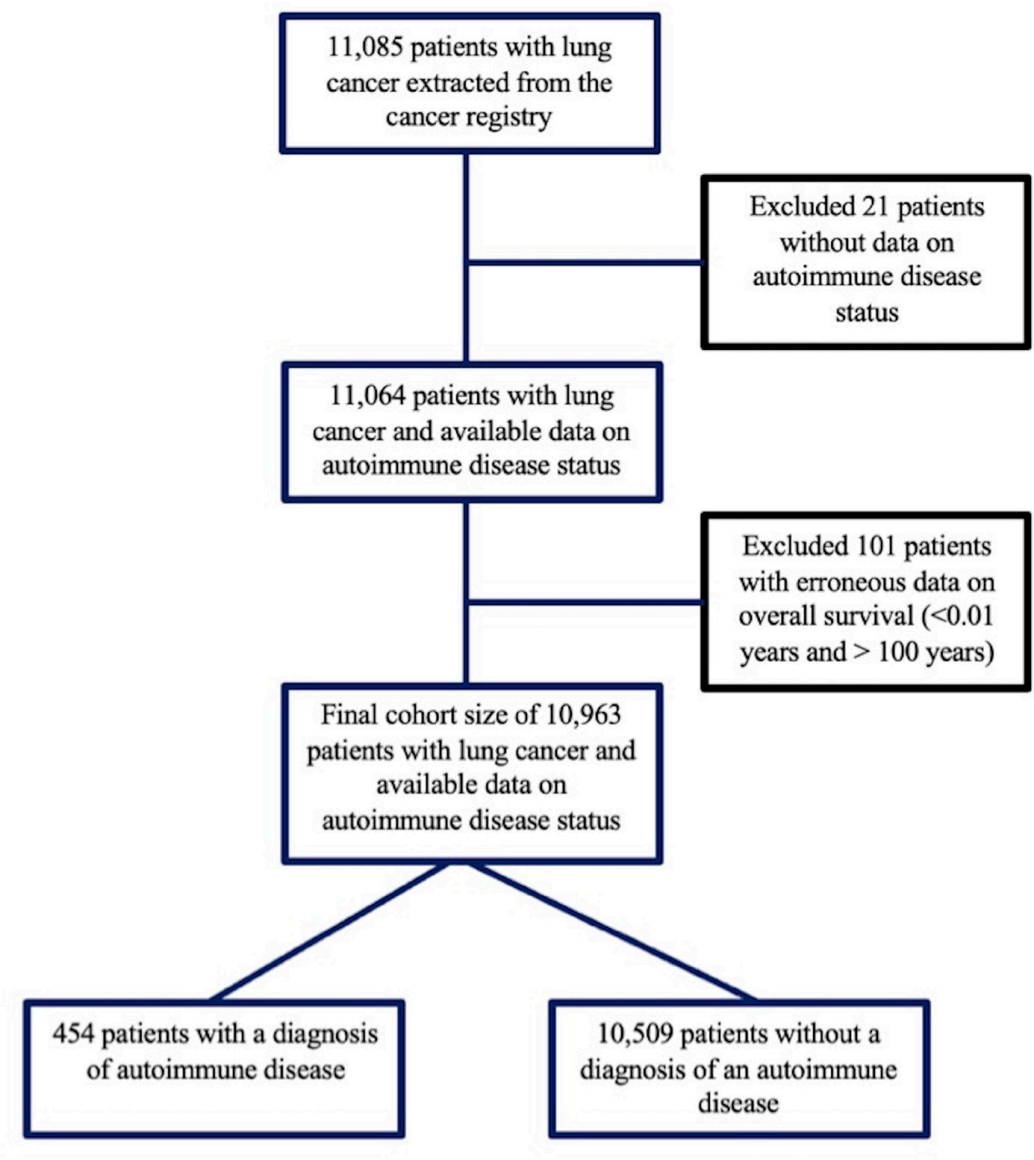
Flow diagram illustrating the cohort selection.

**Figure 2:**
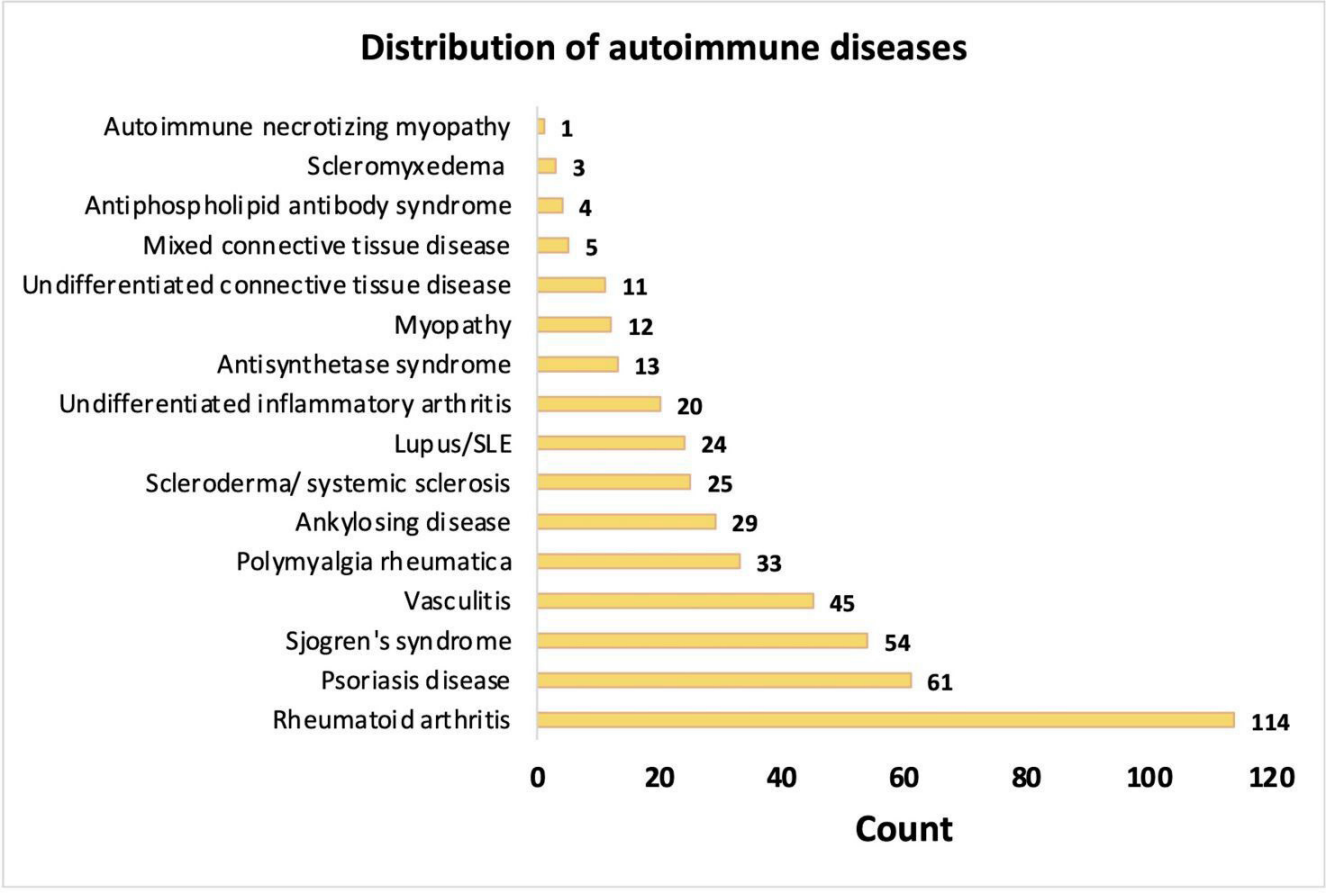
Distribution of autoimmune diseases. SLE, systemic lupus erythematosus.

Data extraction from the cancer registry resulted in 11 085 patients with lung cancer. Patients with missing data regarding their ARD status were excluded from this cohort (n=21), as well as patients with an OS of less than 0.01 years and more than 100 years were excluded from this cohort (n=101). A total of 10 963 patients were included in all subsequent analyses. ARD was present in 3.6% of patients (n=454) (figure 1). The cohort’s characteristics are summarised in table 1. The mean age at diagnosis was 69±10 and 67±12 in patients with and without ARD (p=0.02). Around 80% (n=256) of patients with ARD were female versus 51.7% (n=5444) of patients without ARD (p<0.001). There was no significant difference in the distribution of white, African American and Asian patients across both groups. Around 12.9% (n=45) and 15.6% (n=1119) of patients with and without an ARD, were never smokers. Among 454 patients diagnosed with an autoimmune disease, 114 (25.1%) were diagnosed with RA, 61 (13.4%) with psoriasis/psoriatic arthritis, 54 (11.9%) with Sjogren’s syndrome, 45 (9.9%) with vasculitis, 33 (7.3%) with polymyalgia rheumatica and 29 (6.4%) with ankylosing spondylitis. The complete distribution of ARD is illustrated in figure 2.

To investigate the predictive role of clinical diagnostic features, we performed simple logistic regressions to identify their association with a history of autoimmune diseases in patients with lung cancer. We identified several unique patient characteristics differentially present in lung cancer patients with ARD. Female sex was found to be statistically significantly associated with a history of ARD (OR: 1.65 (1.34 to 2.03), p=0.001), particularly in patients with RA, systemic lupus erythematosus (SLE), systemic sclerosis, Sjogren’s syndrome and undifferentiated inflammatory diseases as highlighted in online supplemental table 2. Compared with their white counterparts, black patients were around three times more likely to have ankylosing spondylitis and SLE. Limited observations could be made with other races due to smaller sample size. Compared with never smokers, former smokers were more likely to be diagnosed with an autoimmune disease (OR: 1.46, p=0.023).

### Tumour characteristics by autoimmune disease status

Lung cancers were categorised by histology as shown in table 1. About 85% of lung cancer diagnoses were characterised as NSCLC in both groups. In patients without an autoimmune disease, 50.2% (n=5300) of lung tumours were adenocarcinoma, 17.9% (n=1886) were SCC, 10.5% (n=1109) were SCLC and 4.3% (n=454) were large cell neuroendocrine tumours, compared with 51.2% (n=206), 18.9% (n=76), 7.7% (n=31) and 6.0% (n=24), respectively in patients with ARD. While lung histology was not found to be predictive of a positive history of ARD generally, patients with large cell neuroendocrine tumours were more likely to be diagnosed with systemic sclerosis (OR: 4.64, p=0.01), while patients with SCLC were less likely to have Sjogren’s syndrome (OR: 0.16, p=0.08). Patients without ARD were most likely to be diagnosed with stage 4 lung cancer (44.7%, n=3973) compared with 30.7% (n=110) in patients with ARD, while the latter were more likely to be diagnosed with stage 1 lung cancer (36.6%, n=131) compared with 26.9% (n=2388) in patients without ARD (p<0.001). As noted in tables 1 and 2, compared with stage 1 disease, stage 3 and 4 were statistically less likely in patients with a history of ARD (OR: 0.71, p=0.021; OR: 0.51, p value <0.001, respectively), including subgroups of patients with polymyalgia rheumatica, RA, SLE, Sjogren’s syndrome and vasculitis. Patients with ARD were also less likely to be diagnosed with distant metastases in the liver and brain (table 1). Finally, patients with ARD were statistically significantly more likely to undergo cancer-related surgery as a treatment modality (48.5%, n=195 vs 35.6%, n=3757, p<0.001). Interestingly, patients with ARD were also more likely to receive monoclonal antibodies that include immune checkpoint inhibitors (ICIs), compared with their counterparts (19.9%, n=80 vs 9.1%, n=958, p value <0.001) and less likely to receive radiation therapy (25.9%, n=104 vs 30.9%, n=3268, p=0.031). There was no difference in chemotherapy administration between the groups.

To further elucidate the relationship between tumour characteristics in lung cancer patients with and without ARD, we performed multivariable logistic regression analysis adjusting for features such as sex, race, histology, smoking and alcohol history that may confound the association between ARD and lower stage of the oncological disease (table 2). Female sex was associated with higher odds of ARD (p<0.001), particularly in patients with undifferentiated inflammatory disease, psoriatic disease and vasculitis (online supplemental table 2). Importantly, after controlling for these potentially confounding factors, stage 4 disease was associated with lower odds of having an autoimmune disease (OR: 0.58, p<0.001), particularly in patients with ankylosing disease, polymyalgia rheumatica, RA, SLE, psoriatic disease and vasculitis (online supplemental table 2). This remained true on grouping of ARDs; inflammatory joint diseases and lupus/Sjogren’s disease were less likely to be associated with advanced stage lung cancer (online supplemental table 3). Importantly, among lifestyle habits, alcohol use was associated with lower risk of ARD which was particularly seen in patients with scleroderma, while smoking history was not associated with risk of ARD except in patients with psoriasis (online supplemental table 2).

### Survival outcomes

To understand survival outcomes in patients with ARD, we applied the Kaplan-Meier method to compare the OS of lung cancer patients with and without ARD. Across the total cohort, lung cancer patients with autoimmune diseases had a significantly prolonged OS compared with their counterparts without autoimmune diseases (median OS: 7.11 years vs 1.7 years). The median OS was evaluated within each stage category. Even after controlling for the fact that patients with ARD are more likely to be diagnosed with early stage disease, the OS remained consistently prolonged. For example, the median OS for patients with stage 1 lung cancer and ARD was not reached compared with a median OS of 7.3 years in patients without ARD. Similarly, patients with stage 4 disease and ARD had a median OS of 1.2 years compared with 0.7 years (p=0.016). The survival benefit in the advanced stage setting was observed across several autoimmune diseases such as ankylosing spondylitis (log-rank p=0.03), RA (log-rank p=0.01), psoriasis (log-rank p=0.003), vasculitis (log-rank p=0.01), Sjogren’s disease (log-rank p=0.03) (data not shown). Additionally, independent of the treatment modality, patients with ARD had a prolonged OS. Notably, lung cancer patients with ARD who have received monoclonal antibodies derived significant clinical benefit; median OS 3.8 years compared with 2.3 years (p=0.02).

**Figure 3:**
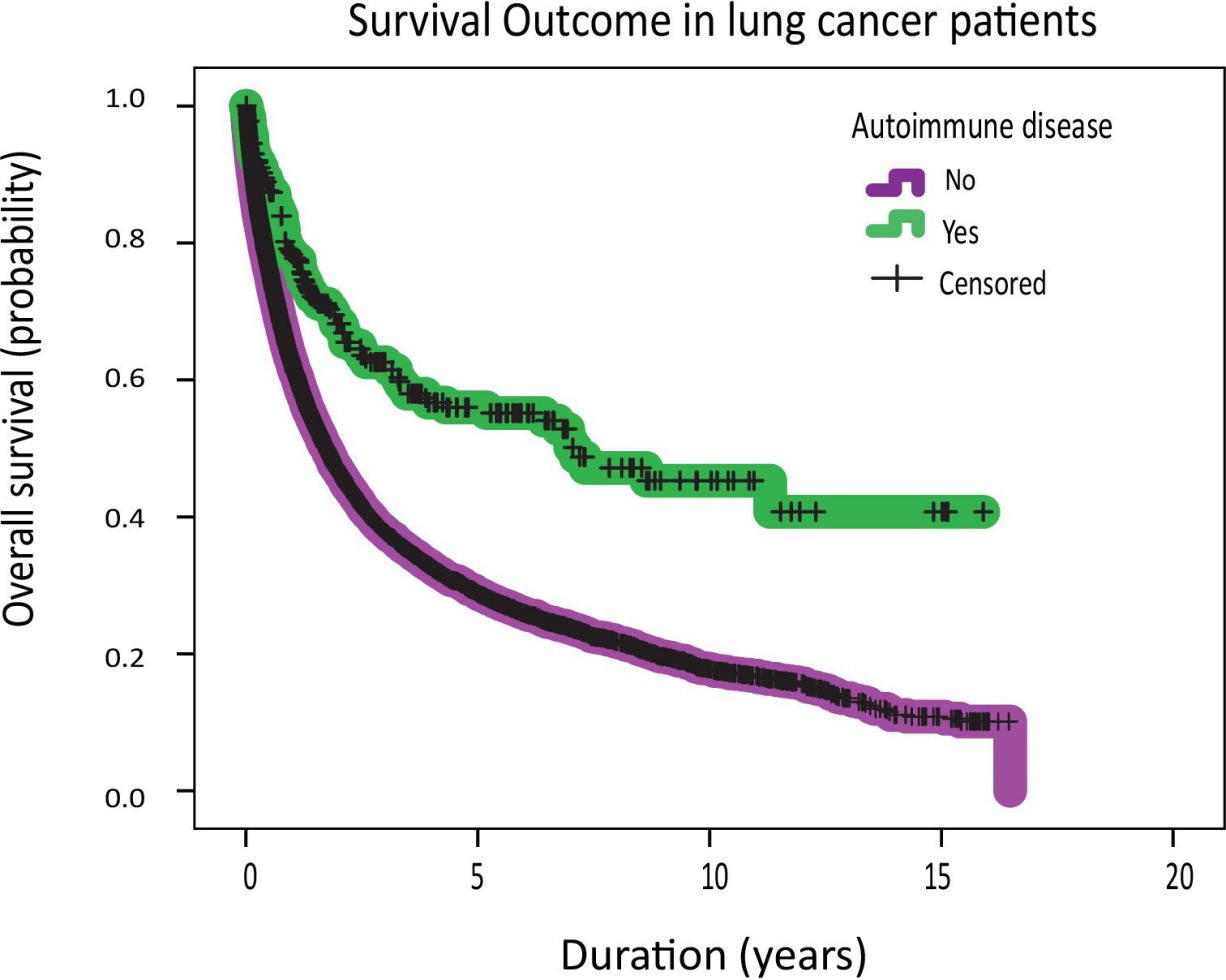
Overall survival among lung cancer patients with and without autoimmune diseases.

Finally, a Cox regression analysis controlling for clinically meaningful variables such as age, stage of the lung cancer diagnosis, smoking history, alcohol history and the different treatment modalities was performed. Interestingly, we observed a survival benefit among lung cancer patients with ARD with an HR of 0.74 (p value =0.003) (table 3, figure 3). More granularly, this was notably seen in patients with inflammatory joint diseases, particularly psoriasis and RA, while patients with systemic sclerosis and myositis had a worse HR of 1.67 (online supplemental tables 4 and 5). Compared with never smoking, a history of smoking was associated with a worse OS (table 3).

**Table 3:** Multivariate Cox regression analysis for overall survival

	HR	95% CI	HR adjusted	95% CI
Autoimmune disease	0.49	0.41 to 0.58	0.74	0.61 to 0.90
Age at diagnosis	1.0	1.01 to 1.02	1.01	1.00 to 1.01
Smoking history	1.71	1.55 to 1.88	1.71	1.53 to 1.90
Alcohol history	1.05	0.98 to 1.12	0.98	0.91 to 1.05
Clinical stage				
Stage 2	1.73	1.51 to 1.97	2.06	1.72 to 2.46
Stage 3	2.89	2.64 to 3.15	3.16	2.75 to 3.63
Stage 4	5.95	5.50 to 6.45	6.50	5.69 to 7.42
Surgery	0.23	0.22 to 0.25	0.50	0.45 to 0.55
Chemotherapy	1.35	1.28 to 1.42	0.61	0.56 to 0.67
Immunotherapy	0.76	0.69 to 0.84	0.54	0.48 to 0.55
Radiation therapy	1.22	1.16 to 1.29	0.9	0.84 to 0.97

The adjusted OR were controlled for age, stage of the lung cancer diagnosis and the different treatment modalities.

### Impact of immunotherapy and immunosuppressive therapy

Importantly in our study, since ‘immunotherapy’ in the institutional registry does not exclusively include ICIs, we evaluated the distribution of monoclonal antibodies before and after 2015 when ICIs became approved for use in lung cancer. In our study, around 96% of monoclonal antibodies were administered after 2015. Patients with ARD were more likely to receive monoclonal antibodies than patients without ARD (19.9%, n=80 vs 9.1%, n=958; p<0.05). Looking at the OS of patients who have received monoclonal antibodies (n=1038), patients with ARD were more likely to have a prolonged median OS (3.8 years vs 2.3 years, p value: 0.02).

When looking at the effect of glucocorticosteroids on clinical outcomes, we note that 84% (n=320) of lung cancer patients with ARD received steroids, compared with 41.1% (n=4337) of patients without ARD (p=0.05). Out of 80 individuals with ARD who received monoclonal antibodies, 61 individuals with ARD had steroid therapy prior to therapy with monoclonal antibodies. To evaluate the effect of glucocorticoids on the clinical benefit of monoclonal antibodies (n=1038), we performed a Cox regression model to evaluate the survival outcomes among patients with and without ARD who received immunosuppressive therapy. Patients with ARD who received monoclonal antibodies had a significantly reduced HR of death compared with their counterparts (HR: 0.6, p=0.012), independent of glucocorticoid use (HR: 1.02, p=0.83).

## Discussion

Patients with lung cancer and ARD represent an important population, particularly in the setting of increased use of immunotherapy. To the best of our knowledge, this is the first study to evaluate tumour characteristics at diagnosis and survival outcomes in a large retrospective cohort of lung cancer patients with and without ARD. We observed a prevalence close to 4% of rheumatic ARD among patients with lung cancer, consistent with the general population.^1^ Patients with ARD were less likely to have stage 4 lung cancer at diagnosis and less likely to have distant metastases. They also had improved OS even when controlling for stage of cancer and treatment modalities used. Finally, in the group of patients treated with immunotherapy, patients with ARD had better OS; this association persisted when controlling for glucocorticoid use among the groups.

Previous studies established the increased occurrence of malignancies in cohorts of patients with ARD,^19 20^ potentially due to shared risk factors,^21^ aberrant immune dysregulation, chronic inflammatory tissue states^22^ and the use of immunomodulatory therapy.^23^ Several clinical characteristics were identified as potential risk factors associated with the development of cancer in a large cohort of patients diagnosed with scleroderma^18^ including but not limited to autoantibody status, age at onset of ARDs and race. However, data on cancer characteristics and outcomes and data specific to patients with lung cancer remain scarce. In our study as predicted, female patients with lung cancer were more likely to have autoimmune diseases. While a positive smoking history was not associated with an increased risk of ARDs in our cohort of patients with lung cancer, it contributed negatively to the OS, which is strongly associated with poor prognosis in patients with lung cancer.^24^ Importantly, alcohol use was found to be negatively associated with ARD which could reflect dietary and lifestyle changes that patients with ARDs undertake. The favourable cancer features at diagnosis in patients with ARD, such as stage 1 disease, and lower rate of distant metastases (brain, liver and bone), are of clinical importance. Patients with earlier stage disease have better survival outcomes. Our findings are consistent with previous retrospective studies, in which patients with ARD were more commonly diagnosed with early-stage lung cancer compared with patients without ARD.^25–27^ This could result from higher healthcare usage due to close medical follow-up among patients with previously established ARD and early detection of cancer. Specifically, many ARDs are associated with interstitial lung disease or other pulmonary conditions, and patients may receive serial CT scans of the chest for monitoring. Interestingly, we observe no significant differences in lung cancer histology between patients with and without ARD.

A novel finding of this study was the superior OS among patients with ARD compared with their lung cancer counterparts without ARD, independent of clinical cancer staging. Previous studies have reported conflicting observations where patients with ARD had worse or equal survival outcomes to those without ARD.^14 15 28^ The median survival in lung cancer patients with RA was not statistically significantly worsened in one retrospective cohort study^28^ while in a smaller but more recent study, mortality was higher in patients with RA and lung cancer when compared with patients who did not have RA.^11^ In a study of the Swedish population, ARD status did not influence OS for lung cancer with the exception of small cell lung cancer where survival was worse in ARD patients.^14^ In our study, compared with patients with lung cancer without ARD, patients with ARD consistently derived clinical benefit from all treatment modalities. There are several possible explanations for this difference. First and most importantly, patients with ARD may have improved access to healthcare and/or health literacy leading to improved survival. However, in our retrospective study, we extract clinical tumour characteristics and outcomes from the cancer registry. Data on patients’ history of 20 ARDs were extracted from the electronic medical record and did not include the date at diagnosis of the ARD. Therefore, due to the nature of our retrospective study, the findings of improved OS can potentially be the results of lead time bias. Alternatively, we also hypothesise that the survival benefit observed in patients with ARD could suggest that the presence of tissue-specific autoimmune responses in patients with ARD lead to improved antitumour immune responses. Future prospective studies accounting for lead time bias will be needed to further understand the observed improved OS in patients with ARD. Additional studies evaluating the relationship between autoreactive T cells/autoantibodies and tumour antigens in patients with lung cancer and ARD can determine how relevant the second mechanism is to clinical outcomes.

Several studies investigated the implications of immunotherapy in lung cancer patients with ARD.^25 29^ In our study, a reduction in HR for death among patients who received immunotherapy (not limited to ICIs) was observed independent of glucocorticoid use. Previously, in a small retrospective study of 112 patients with ARD, the median progression-free survival was shorter in patients receiving immunosuppressive therapy on treatment initiation with ICIs.^30^ There remain however, significant knowledge gaps in understanding and deciphering the clinical and tumour characteristics of patients with ARD who would most benefit from immunotherapy. Several studies have demonstrated that immune-related adverse events from ICIs have distinct biological features such as cytokines levels, MHC genes, as compared with the conventional autoimmune diseases.^31 32^ Flares of the underlying autoimmune disease can occur, however, in about half of patients with ARD who are treated with ICIs.^17^ Future prospective trials of patients with ARD and lung cancer will clarify any potential efficacy benefits or safety issues for this specific group of patients.

Our study has some limitations. While this study includes around 20 different types of ARD, the small sample size for individual categories of ARD limits the generalisability of our findings to each ARD. Importantly, additional studies are needed to adjust for the lead time bias that results from a higher healthcare usage in patients with ARD. In addition, due to the use of a large database, incomplete assessment of clinically relevant end points limits the comprehensive understanding of cancer-related outcomes in this patient population, such as the lack of knowledge on occurrences of immune-related adverse events, data on cancer specific mortality, and on tumour genotype profiling of NSCLC. Importantly, this large database also possesses variations in defining immunotherapy as it includes ICIs, monoclonal antibodies and other types of immune-modulating agents. It is also important to note that the high rates of ICIs use in the ARD group studied in this cohort could represent a selection bias toward a group of patients with clinically less severe rheumatic disease, who might therefore have a more favourable response to immune checkpoint blockade.

This study leverages a large retrospective cohort and adds to the pool of knowledge regarding cancer-related outcomes in this unique patient population. In this study, we identify a clinical benefit across all treatment modalities in patients with ARD, even among patients with advanced stage disease. Importantly, we show that patients with ARD who received immunotherapy perform clinically well.

## Conclusion

This large retrospective cohort adds to the literature regarding several patient and tumour-specific features associated with lung cancer in patients with ARD. While our study identifies a survival benefit in patients with ARD compared with their counterparts, independent of lung cancer stage and treatment modalities, additional in-depth studies are needed to account for lead-time bias. In addition, more studies are needed to investigate with more granularity the clinical, serological and genomic determinants of patients living with lung cancer and autoimmune diseases and help guide individualised treatment opportunities.

**Key messages**

- Conflicting data exists about whether lung cancer outcomes differ in patients with autoimmune rheumatic diseases as compared to the general population.
- In this study, patients with autoimmune rheumatic disease and lung cancer had smaller tumours, lower disease stage at presentation and better overall survival.
- Additional studies raising the question of whether underlying immunological or genomic differences, increased health surveillance, or a combination of both lead to the above findings are needed.

## Data Availability

Data are available upon reasonable request. Deideintified data will be available upon reasonable request.
